# Chemotherapy-Induced Unconjugated Hyperbilirubinemia Complicated by Other Trigger Factors in a Child with T-Cell Acute Lymphoblastic Leukaemia and *UGT1A1* Mutation-Associated Gilbert Syndrome

**DOI:** 10.3390/curroncol32020091

**Published:** 2025-02-07

**Authors:** Mohammad Shukri Khoo, Sharifah Naiema Jamalullail, C-Khai Loh, Sie Chong Doris Lau, Hamidah Alias

**Affiliations:** 1 Department of Paediatrics, UKM Specialist Children’s Hospital, Faculty of Medicine, The National University of Malaysia, Kuala Lumpur 56000, Malaysiadoris@ppukm.ukm.edu.my (S.C.D.L.); 2 Clinical Research Unit, Department of Paediatrics, UKM Specialist Children’s Hospital, The National University of Malaysia, Kuala Lumpur 56000, Malaysia

**Keywords:** Gilbert syndrome, T-cell acute lymphoblastic leukaemia, hyperbilirubinemia, chemotherapy

## Abstract

Gilbert syndrome (GS) is an inherited disorder characterised by unconjugated hyperbilirubinemia due to a deficiency in hepatic UDP-glucuronosyltransferase 1A1 (*UGT1A1*) enzyme activity, responsible for bilirubin glucuronidation. This results in decreased bilirubin conjugation and excretion, leading to elevated serum unconjugated bilirubin levels. In T-cell acute lymphoblastic leukaemia (T-ALL), treatment typically involves intensive chemotherapy regimens that include agents metabolised by the liver, requiring careful consideration of liver function and bilirubin metabolism in patients with concurrent GS. We present the case of a 15-year-old male who was diagnosed with T-ALL and treated with a chemotherapy regimen following the modified Dutch Child Oncology Group ALL-9 (High Risk) protocol. Concurrently, the patient was observed to have persistent unconjugated hyperbilirubinemia aggravated by infection and fasting despite normal to mildly deranged liver function, which was initially assumed to be attributed by 6-Mercaptopurine (6-MP). Further workup confirmed a diagnosis of GS based on clinical history, laboratory findings, and genetic testing. We recommend performing a genetic analysis of *UGT1A1* in patients presenting with chemotherapy-induced hyperbilirubinemia with no signs of liver impairment. This aims to prevent unnecessary alterations in chemotherapy regimens that could potentially increase the risk of relapse.

## 1. Introduction

**Figure 1:**
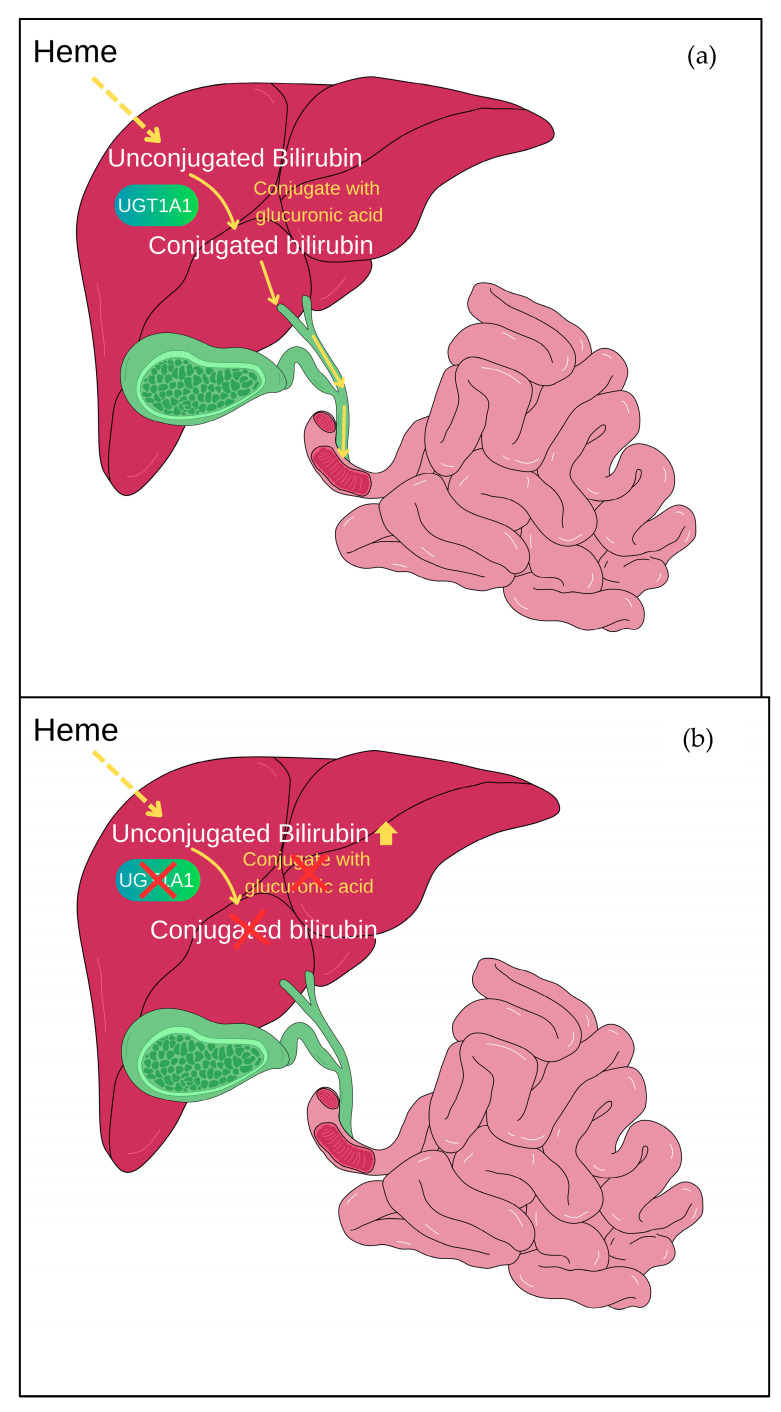
Bilirubin glucuronidation mediated by *UGT1A1*. (**a**) *UGT1A1* aids in the conjugation of bilirubin with glucuronic acid, which is known as glucuronidation. This conjugation makes bilirubin water-soluble, facilitating its elimination from the body. In normal situations, after glucuronidation, the conjugated bilirubin is transported through bile into the intestines and subsequently expelled from the body through faeces. (**b**) In Gilbert syndrome, the mutation of the *UGT1A1* gene leads to reduced activity of the *UGT1A1* enzyme, causing an accumulation of unconjugated bilirubin in the body.

Gilbert syndrome (GS) is a typically inherited benign condition affecting about 5 to 10% of the population [1]. The key findings associated with GS (Table 1) include elevated levels of indirect serum bilirubin, typically around 85% from the normal baseline, with concentrations usually below 4 mg/dL [2]. In rare cases, bilirubin levels may be excessively high, necessitating treatment with phenobarbital [3,4]. Liver function tests including albumin levels, and prothrombin time are typically within normal ranges. Liver biopsy results also typically show no abnormalities. GS is known to result from mutations involving seven to eight thymine adenine (TA) repeats in the promoter region of the bilirubin uridine-5-diphosphate (UDP)-glucuronosyltransferase gene (*UGT1A1*) [5], leading to impaired bilirubin glucuronidation (Figure 1) due to the production of a less efficient form of the *UGT1A1* expression by 30% [6]. When red blood cells undergo haemolysis, they release unconjugated bilirubin into the bloodstream. This bilirubin is then bound to carrier proteins and transported to the liver. Within the liver, an enzyme called glucuronyl transferase facilitates the conjugation process, where bilirubin is converted into a water-soluble form for excretion into bile. Nevertheless, individuals with GS have a deficiency in this glucuronyl transferase enzyme, leading to the impaired conjugation of bilirubin in the liver and manifestation as jaundice. Jaundice in patients with GS tends to manifest during periods of fasting, dehydration, physical exertion, stress, infections, and menstruation [2].

T-cell acute lymphoblastic leukaemia (T-ALL) accounts for between 12% and 15% of newly diagnosed cases of ALL in paediatric patients [7]. Currently, these patients are usually treated with risk-based new multiagent chemotherapy regimens that are more aggressive, resulting in consistent improvement in paediatric T-ALL outcomes, though resulting in greater toxicity. Some chemotherapy agents such as daunorubicin, pegaspargase, and vincristine can occasionally result in elevated levels of unconjugated bilirubin, even in the absence of liver impairment. 6-mercaptopurine (6-MP), an antimetabolite drug used in treating ALL along with other chemotherapeutic agents that inhibit DNA and RNA synthesis, can decrease the production of the *UGT1A1* enzyme in hepatocytes [8]. When hyperbilirubinemia occurs under these circumstances, chemotherapy may need to be halted according to the treatment protocol. However, patients carrying genetic mutations in the *UGT1A1* gene can result in just a temporary increase in unconjugated bilirubin levels during chemotherapy. Therefore, a molecular diagnosis of GS could be crucial for clinicians in cases of persistent unconjugated hyperbilirubinemia during treatments. This diagnosis suggests that the elevated bilirubin levels may not solely be attributable to chemotherapy-induced effects. Therefore, reducing doses of these drugs may not be necessary.

In this case report, a young child of Southeast Asian ethnicity with T-cell ALL, who demonstrated reversible non-haemolytic indirect hyperbilirubinemia during maintenance chemotherapy, was found to have a *UGT1A1* polymorphism consistent with GS.

## 2. Case Presentation

The patient, a 15-year-old male of Malay descent, was diagnosed with T-ALL at the age of 13 years following a two-week history of progressively enlarging painless neck swelling. Physical examination revealed bilateral multiple cervical lymphadenopathies, with the largest node measuring 5 cm x 6 cm, non-tender, and not fixed to the skin or underlying structures. Hepatosplenomegaly was noted, with the liver measuring 8 cm and the spleen 11 cm below the costal margin. Laboratory investigations demonstrated hyperleukocytosis with a white blood cell count of 328 × 10^9^/L and leucoerythroblastic changes, characterised by 93% blasts. Bone marrow examination confirmed the diagnosis of T-cell ALL. The patient was initiated on chemotherapy following the modified Dutch Childhood Oncology Group ALL-9 (High Risk) protocol that consists of dexamethasone, high-dose methotrexate (MTX), 6-MP, triple intrathecal, vincristine, daunorubicin, and cytarabine/cyclophosphamide, resulting in the achievement of morphological remission.

Throughout the treatment courses, he experienced episodes of febrile neutropenia and developed vincristine-induced myopathy and ileus, which responded to appropriate management. While receiving the ninth cycle of maintenance chemotherapy, he developed fever, jaundice, and lethargy. There was no history of abdominal pain, bleeding tendency, gastrointestinal symptoms, or pruritic skin. On examination, he appeared non-toxic, with a temperature of 38.5 °C, pallor, and jaundice. His hydration status was fair, and there were no clinical signs of infection noted. The liver was palpable at 2 cm and non-tender. Laboratory investigations revealed unconjugated hyperbilirubinemia, but no evidence of liver dysfunction was observed at that point. It was initially concluded that the hyperbilirubinemia was likely caused by the oral chemotherapy, 6-MP. The dose of oral 6-MP was adjusted, and liver function was closely monitored. Following the dose modification, serum bilirubin levels decreased but did not normalise.

**Figure 2:**
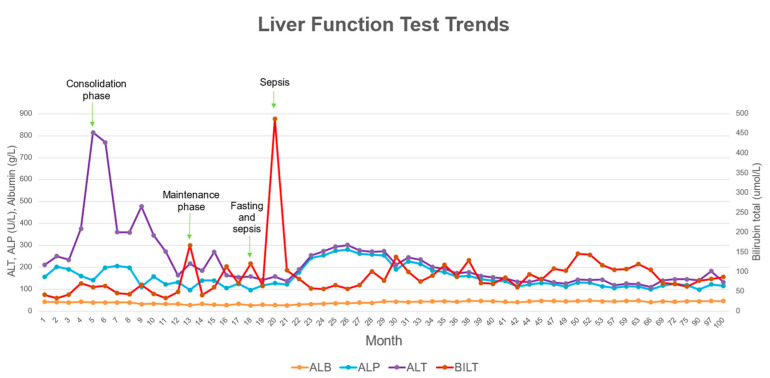
Liver function test trends throughout patient’s treatment period.

Later, he presented with fever and abdominal distension after fasting for two days during Ramadan (a holy month observed by Muslims where they fast from sunrise to sunset). He was neutropenic and diagnosed with *Escherichia coli* septicaemia, which resolved with antibiotics. During this episode, he developed severe unconjugated hyperbilirubinemia, and the 6-MP chemotherapy was temporarily halted until bilirubin levels decreased. He experienced another episode of sepsis two months later, this time caused by *Acinetobacter baumanii*, during which his serum bilirubin rose to 487 μmol/L. The jaundice was observed to worsen during both infection and fasting states. The trend in his serum bilirubin levels and other liver function tests are illustrated in Figure 2.

**Figure 3:**
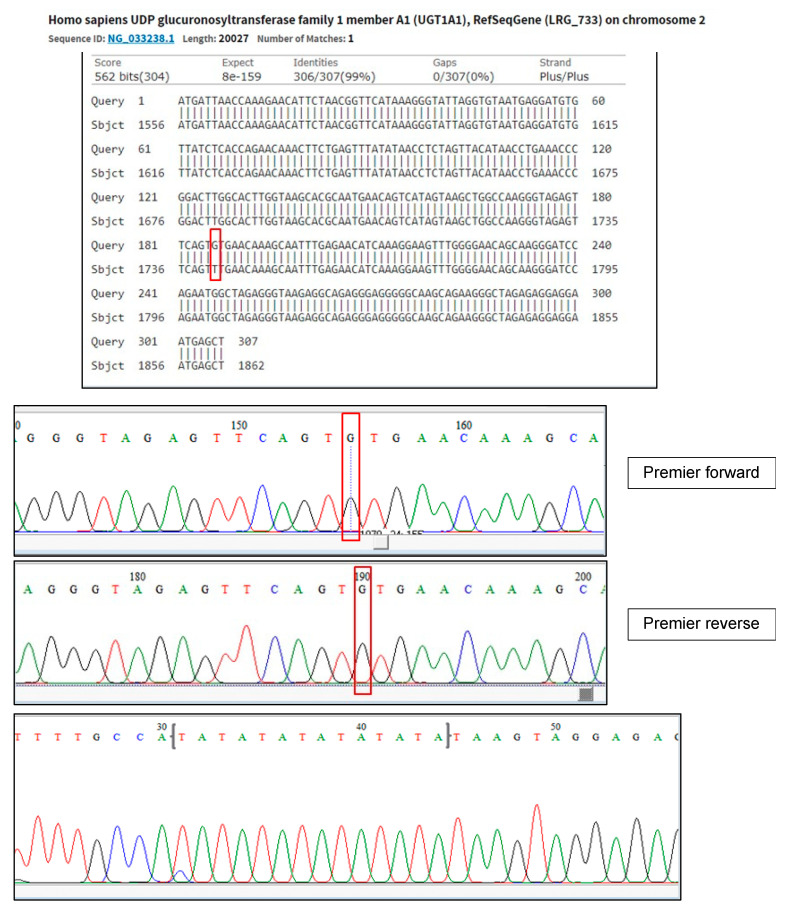
Genetic test confirms the presence of mutation c.-3279T>G (rs4124874), NG_033238.1: g.1741T>G and genetic variant A(TA)_7_TAA at TATA BOX (rs34983651). Red boxes highlight pathogenic mutation in this genetic test when identifying *UGT1A1* mutation.

Further investigations ruled out infective causes, including hepatitis, metabolic disorders, haemolysis, and autoimmune conditions. Serial ultrasound and computer tomographic (CT) scans of the abdomen showed hepatosplenomegaly and early signs of portal hypertension. Liver biopsy revealed periportal hepatitis with fibrosis stage 4, likely induced by MTX. Due to persistent unconjugated hyperbilirubinemia despite normal to mildly deranged liver function, deficiency in the *UGT1A1* enzyme was considered among the differential diagnoses. Subsequently, genetic testing confirmed two *UGT1A1* variants, the c.-3279T>G hotspot (G/G) and A(TA)7TAA variant in the TATA box, consistent with a diagnosis of Gilbert syndrome (Figure 3). Parental genetic testing was not performed; therefore, biparental inheritance could not be confirmed.

## 3. Discussion

GS, when combined with conditions like thalassaemia, glucose-6-phosphate dehydrogenase (G6PD) deficiency, spherocytosis, and ALL can increase the risk of severe hyperbilirubinemia [3]. Only a handful studies have investigated the correlation between GS and chemotherapy in leukaemia patients [9,10,11,12,13]. Some hypothesised that chemotherapy-induced nausea, episodes of febrile neutropenia, and reduced appetite exacerbate hyperbilirubinemia in these patients. Individuals with GS often experience varying levels of serum bilirubin that can worsen with caloric loads, with fasting being a significant trigger for hyperbilirubinemia. Additionally, chemotherapy medications commonly provoke emesis and loss of appetite in patients with ALL, also leading to reduced caloric intake.

More recent studies have suggested instead that the metabolism of chemotherapy itself contributes to hyperbilirubinemia. This is rather important because in some chemotherapy protocols, elevated bilirubin levels above specific thresholds determine adjustments to the timing or dosage of various chemotherapy drugs [14,15]. This situation can lead to delays in treatment, reductions in dosage, or even discontinuation of treatment, all of which can potentially affect the overall outcomes for these patients.

In our patient, the clinical presentation of fluctuating unconjugated hyperbilirubinemia prompted us to investigate the status of the UGT1A1 gene. The discovery of two *UGT1A1* variants, the c.-3279T>G and A(TA)7TAA variants, combined with ongoing chemotherapy, posed a challenge for our team in making treatment decisions. The findings of homozygous variants c.-3279T>G and A(TA)7TAA in our patient support the pathogenesis of GS as reported by Maruo et al. [16]. Further, it is crucial for clinicians to understand the pharmacokinetics of specific chemotherapeutic agents metabolised by *UGT1A1*, as this may necessitate dose adjustments and monitoring in the presence of GS, due to uncertain risks of increased toxicity or adverse effects in these cases. The problem comes from the reliance of dose adjustment guidelines for most chemotherapy drugs on liver function, with serum bilirubin levels being the parameter most commonly used to determine the dose adjustment [17]. However, in GS patients, liver function typically remains normal despite elevated bilirubin levels. So, in the case of GS, this modification might not be necessary because this means that their ability to metabolise most chemotherapy agents remains intact despite elevated bilirubin levels [15]. Nevertheless, more reports and further research in relation to chemotherapy in GS patients are needed because of the limited number of patients in published studies, and the scarce number of studies make it challenging to observe potentially significant differences in how these chemotherapy drugs are metabolised between individuals with GS and its potential harmful effects.

In paediatric ALL, certain chemotherapy agents undergo glucuronidation, potentially leading to impaired drug clearance and increased drug toxicity or adverse events in GS patients. Therefore, the determination of hepatic dysfunction using bilirubin alone is insufficient, and other liver-related blood investigations including alanine and aspartate transaminases, prothrombin time, and albumin, as well as the patient’s clinical status, need to be taken into consideration. A study by Berrueco et al. [11] observed that serum bilirubin levels in patients with GS were significantly elevated across all treatment phases in paediatric ALL, particularly in those who underwent more intensive protocols due to increased depletion of asparagine caused by a different schedule of L-asparaginase administration, where liver toxicity and hyperbilirubinemia commonly occur as side effects of this treatment. However, this study concluded that no significant differences in toxicities, overall survival, or progression-free survival outcomes were observed between patients with GS and those without GS. A prospective study on paediatric ALL and GS [18] found that slower MTX clearance was associated with increased gastrointestinal side effects and hyperbilirubinemia in the patients. Patients carrying the *UGT1A1* 7/7 genotype, exhibited a notably reduced average MTX clearance in contrast to those with other *UGT1A1* genotypes. This genotype was also identified as a significant predictor of hyperbilirubinemia even after accounting for MTX clearance levels. Although higher MTX exposure and higher bilirubin values were noted in patients with GS, the incidence of adverse effects also did not appear to be different in this population compared to those without GS.

Therefore, after considering all other factors, our patient with the homozygous variants c.-3279T>G and A(TA)7TAA of *UGT1A1* and unconjugated hyperbilirubinemia during ALL chemotherapy, without liver dysfunction, is unlikely to have hyperbilirubinemia attributable to chemotherapy adverse effects; rather, it is likely due to GS. However, chemotherapy dosages like MTX and 6-MP might need to be reduced if concurrent infection occurs in the patient.

The routine mutational screening of the *UGT1A1* gene in cancer patients remains a topic of debate. Further, a careful analysis of the specimen type tested for *UGT1A1* variants is important to confirm any constitutional variants in different tissue types like fibroblasts to differentiate them from variants that may arise due to clonal- or chemotherapy-related scenarios. While some authors advocate for this analysis to personalise treatment in certain cancer patients, others argue that chemotherapy adjustments are unnecessary in GS. Only a few chemotherapeutic agents, like irinotecan and sorafenib, have well-documented toxicities [19] that justify genotyping before treatment initiation, but this is not necessary for ALL paediatric because none of the protocols use these two chemotherapy drugs. However, concerning cases where these ALL patients undergo haematopoietic cell transplant, a study by Mcdonald et al. [20] observed that the overall mortality and non-relapse mortality by day 200 post-transplant were notably poorer in patients with GS who underwent busulfan-containing myeloablative conditioning regimens.

## 4. Conclusions

We suggest conducting a genetic analysis of *UGT1A1* for patients experiencing chemotherapy-induced hyperbilirubinemia without any signs of liver dysfunction to avoid unnecessary changes in chemotherapy that could increase the risk of relapse. Once such a mutation is confirmed, therapy can proceed with minimal concern that chemotherapy is inducing liver damage in the absence of trigger factors like infection. That said, other causes of unconjugated hyperbilirubinemia should be ruled out first, as GS is a diagnosis of exclusion.

## Figures and Tables

**Table 1:** Key findings of tests associated with Gilbert syndrome.

Test	Result(s)
Serum bilirubin	Total bilirubin– mildly elevated Conjugated bilirubin – normal Unconjugated bilirubin – elevated
Faecal urobilinogen	Decreased
UFEME ^a^	No bilirubin
Liver function tests (ALT ^b^, AST ^c^, ALP ^d^, GGT ^e^, albumin, total protein, and PT ^f^ )	Normal
Liver biopsy	Normal

^a^ Urine Full Examination and Microscopic Examination, ^b^ alanine transaminase, ^c^ aspartate transaminase, ^d^ alkaline phosphatase, ^e^ gamma-glutamyltransferase, ^f^ prothrombin time.

## Data Availability

The data presented in this study are available in this article.
